# Supplementary material for: Mechanistic basis of L-lactate transport in the SLC16 solute carrier family
Source: Nat Commun. 2019 Jun 14;10:2649. doi: 10.1038/s41467-019-10566-6 (PMC6573034; doi:10.1038/s41467-019-10566-6)
Supplement: Supplementary file 1 — Supplementary Information [file 41467_2019_10566_MOESM1_ESM.pdf]

Mechanistic basis of L-lactate transport in the SLC16 solute carrier family

Bosshart et al.

Supplementary Table 1 Data collection and refinement statistics for SfMCT

**Data collection and processing**	High-Resolution	Phasing[a]	SeMet[b]	L-lactate[c]
Beamline	X06SA, Swiss Light Source - SLS	X06SA, Swiss Light Source - SLS	X06SA, Swiss Light Source - SLS	X06SA, Swiss Light Source - SLS
Detector	Eiger 16M	Eiger 16M	Eiger 16M	Eiger 16M
Wavelength (Å)	1.0	1.0	0.9795	1.0
Space group	$P2_12_12$	$P2_12_12$	$P2_12_12$	$P2_12_12$
Unit-cell: a, b, c (Å); $\alpha = \beta = \gamma$ (°)	106.8, 200.5, 64.6; 90	108.3, 200.1, 65.3; 90	106.3, 200.1, 65.4; 90	103.4, 200.0, 63.3; 90
Anisotropy direction[d]				
overall (Å)	**2.54**	**2.58**	**3.17**	**2.69**
along h axis (Å)	2.85	2.84	3.25	2.84
along k axis (Å)	**2.30**	**2.42**	**2.95**	**2.50**
along l axis (Å)	3.13	3.12	4.19	3.43
Resolution (Å)[e]	53.26-2.30 (2.54-2.30)	53.85-2.42 (2.59-2.42)	48.68-2.95 (3.19-2.95)	47.52-2.50 (2.68-2.50)
Measured reflections	447,971 (19,461)	4,411,782 (129,209)	1,689,244 (77,851)	1,729,995 (76,740)
Unique reflections	34,625 (1,731)	36,569 (1,816)	19,075 (954)	30,961 (1,341)
Redundancy	12.9 (11.2)	120.6 (71.2)	88.6 (81.6)	55.9 (57.2)
R_{meas}[f]	0.07 (1.7)	0.21 (5.6)	0.156 (6.9)	0.118 (5.1)
$R_{p.i.m.}$[g]	0.03 (0.7)	0.03 (0.9)	0.02 (0.8)	0.02 (0.9)
$CC_{1/2}$[h]	99.9 (59.4)	100.0 (68.2)	100.0 (59.3)	100.0 (59.1)
Mean $I/\sigma(I)$	19.9 (1.7)	44.6 (1.4)	30.7 (1.1)	40.8 (1.3)
Completeness (%)[i]	94.0 (87.1)	94.7 (93.9)	93.3 (85.2)	94.4 (88.1)
**Refinement**				
Resolution (Å)	24.79–2.30			24.83–2.50
R_{work}/R_{free}[k] (%)	21.75/25.41			23.90/25.93
No. of atoms	5932			5771
Protein	5757			5732
Ligands	173			39
Water	2			–
Mean B factor (Å^2)	80.2			115.8
Protein	80.0			115.8
Ligands	86.3			122.5
Water	46.5			–
RMSD				
Bond length (Å)	0.009			0.007
Bond angle (°)	1.156			0.911
Ramachandran plot (%)				
Favored region	98.26			98.66
Allowed region	100			100
Disallowed region	0			0

[a] Datasets from 3 crystals were merged.

[b] Datasets from 2 crystals were merged.

[c] Datasets from 4 crystals were merged.

[d] The anisotropic resolution limits were computed with AIMLESS[1] based on $CC_{1/2} > 0.50$.

[e] These statistics are for data that was truncated by STARANISO software (http://staraniso.globalphasing.org/) to remove poorly measured reflections affected by anisotropy. Values in parentheses are for the highest resolution shell.

[f] R_{meas} as defined by Diederichs and Karplus (1997)[2].

[g] Precision-indicating merging R factor $R_{p.i.m}$ as defined by Weiss (2001)[3].

[h] $CC_{1/2}$ is the Pearson correlation coefficient of two-half data sets as described by Karplus and Diederichs (2012)[4].

[i] The completeness after the anisotropic correction was obtained by least-square fitting an ellipsoid to the reciprocal lattice points at the cut-off surface defined by a local mean $I/\sigma I$ threshold of 1.2, rejecting outliers in the fit due to spurious deviations, and calculating the fraction of observed data lying inside the ellipsoid.

[k] Random 5% reflections from working set were excluded from refinement for R_{free} calculation.

Supplementary Table 2 Sequence of the codon-optimized SfMCT gene

```
ATGGCTGATCAACAAACGACGATGCCGCGCTGGGTGCCGCTGCTGCTGGGTCTGCTGGGTTCAACGACGTGTGGTATGCTGCT
GTATGCGTGGAGTGTGTTTATTAAACCGCTGAACGCAGAATTTGGCTGGTCCCGTGCTGAAATCGCTATGGCGTTTGCCATTT
GCTGTCTGATCTTTGGTCTGATGACCTTCCCGGCAGGCCGTCTGTCAGATAAAATGGGTCCGCGCAAGGTGGTTATGACGGGC
GGTGTCCTGCTGGCCATTGGTTTTATCCTGAGTGGCTTCATTCAGTCCAAATATCAACTGTACATTACCTATGGTGTTATCGC
CGGCTTTGGCGGTGGCATGATTTATCTGCCGCCGATCGCAACCGCTCCGAAGTGGTGGCCGGACCGTCGCGCACTGGCAACGG
GTTTTGCAGTCGTGGGTCTGGGTCTGGGTTCGTTCCTGATGGGTCCGCTGGCCACCTATATTATCGAAAAACCGGGCATGGGT
TGGCGCTACGTCTTTTGGTATTGCGGTGTGGCGATGGGCATTATGGCCCTGATCGCAGGTGCTTTCCTGGAACCGCCGCCGGC
AGGTTGGAAACCGGCTGGCTACACCCCGCCGGCACCGCCGGCGGGTGCGGCCGCACCGAAGGTGACCCGTGATTGGACGTATG
AAGAAGCGAAAGGTGACACGAAGTTTTGGCTGCTGTACCTGGCGTATTTTTGTGGTAGTTTCGCCGGCCTGATGGTGATTGGT
CATCTGGCAGGTTTCGGTCGTGATGCAGGTCTGACCGCAATGGCTGCGGCCGGTGCAGTTAGCTCTCTGGCCTTTAGCAATGC
AGCTACGCGTATCCTGTCTGGCTGGTTCGTTGACAAAATTGGTATCCGCGTCTACTTTGCGGCCCTGTTCGCACTGCAAACCG
CAGCTATGATTGCGATCTTTCAACTGGGTGGCTCAGTTGTCGGCCTGTCGATTGTGGCGATTGTTATCGGTTGGAACTATGGC
GCAATGTTTACCCTGTTCCCGGCTACGTGCCTGCAATTTTACGGTCCGACCGCCCAAGGTTCTAATTATGGCCTGCTGTTTAC
GGCATGTGGTCTGGCAGGTTTCGCTGGTCCGTGGGTGGGTGGCTGGCTGAAAGATACCACGGGCACCTATTACCTGCCGTTTC
TGTGCGCGGCCGCACTGTGTGCACTGGGCACCGCTATCGTTTTCATGACGAAGCCGCCGGAAAAGAAACACGCG
```

Supplementary Table 2 Sequence of the codon-optimized SfMCT gene, which was used for overexpressing SfMCT in *E. coli*.

Supplementary Table 3 Table of primers used in this study

SfMCT-L28A	5’-GAC GTG TGG TAT GCT GGC GTA TGC GTG GAG TGT G-3’
SfMCT-Y119A	5’-CTT TGG CGG TGG CAT GAT TGC TCT GCC GCC GAT CGC AAC-3’
SfMCT-Y119F	5’-GGC GGT GGC ATG ATT TTT CTG CCG CCG ATC GC-3’
SfMCT-L145A	5’-GTT TTG CAG TCG TGG GTG CGG GTC TGG GTT CGT TCC-3’
SfMCT-H250A	5’-GCC TGA TGG TGA TTG GTG CTC TGG CAG GTT TCG GTC-3’
SfMCT-H250F	5’-GCC TGA TGG TGA TTG GTT TTC TGG CAG GTT TCG GTC-3’
SfMCT-R256A	5’-CAT CTG GCA GGT TTC GGT GCT GAT GCA GGT CTG ACC-3’
SfMCT-R256D	5’-GTC ATC TGG CAG GTT TCG GTG ATG ATG CAG GTC TGA CCG C-3’
SfMCT-D257A	5’-CTG GCA GGT TTC GGT CGT GCG GCA GGT CTG ACC GCA ATG G-3’
SfMCT-N276A	5’-CTC TCT GGC CTT TAG CGC TGC AGC TAC GCG TAT C-3’
SfMCT-R280A	5’-CTT TAG CAA TGC AGC TAC GGC TAT CCT GTC TGG CTG G-3’
SfMCT-Y331A	5’-GTT ATC GGT TGG AAC GCT GGC GCA ATG TTT ACC-3’
SfMCT-Y331F	5’-GTT ATC GGT TGG AAC TTT GGC GCA ATG TTT ACC-3’
SfMCT-F335A	5’-GGA ACT ATG GCG CAA TGG CGA CCC TGT TCC CGG CTA C-3’
SfMCT-F359A	5’-CTA ATT ATG GCC TGC TGG CTA CGG CAT GTG GTC TGG C-3’
SfMCT-C362A	5’-GCC TGC TGT TTA CGG CAG CTG GTC TGG CAG GTT TCG C-3’
SfMCT-K377A	5’-GGG TGG GTG GCT GGC TGG CAG ATA CCA CGG GCA CC-3’
SfMCT-K377D	5’-GTG GGT GGG TGG CTG GCT GGA CGA TAC CAC GGG CAC CTA-3’
SfMCT-D378A	5’-GGT GGG TGG CTG GCT GAA AGC GAC CAC GGG CAC CTA TTA CC-3’
SfMCT-Y383A	5’-CTG AAA GAT ACC ACG GGC ACC GCG TAC CTG CCG TTT CTG TGC-3’
SfMCT-Y383F	5’-GAA AGA TAC CAC GGG CAC CTT TTA CCT GCC GTT TCT GTG-3’
5’-EcoRI-SfMCT	5’-GCA GAA TTC ATG GCT GAT CAA CAA ACG ACG-3’
3’-XhoI-SfMCT	5’-GCG CCT CGA GCG CGT GTT TCT TTT C-3’

Supplementary Table 3 Table of primers used to generate SfMCT mutants by site-directed mutagenesis and for generating the pEXT20-SfMCT-3C-10His construct (i.e., 5’-EcoRI-SfMCT and 3’-XhoI-SfMCT).

Supplementary Figure 1 Functional characterization of SfMCT

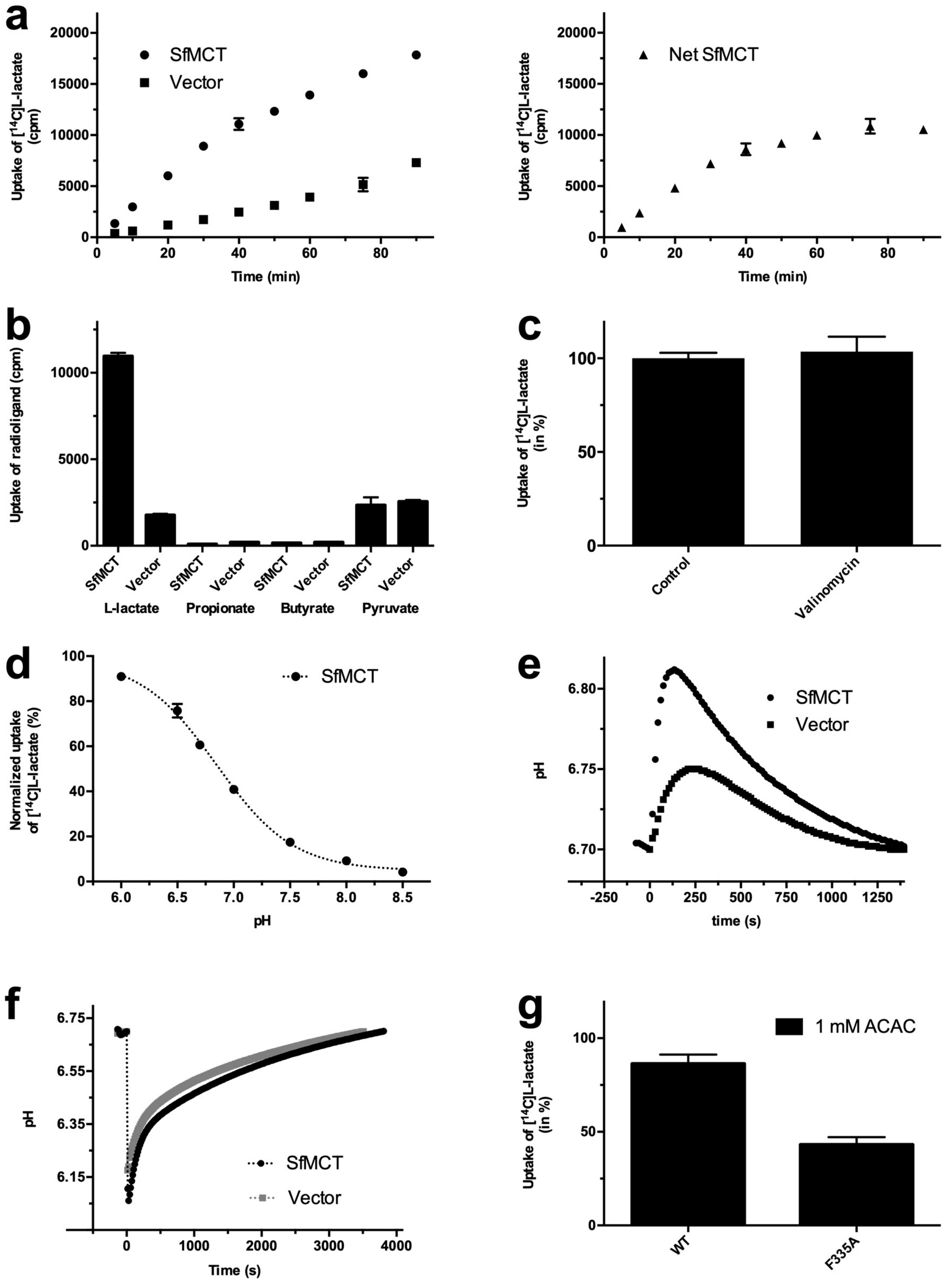

Supplementary Figure 1 Functional characterization of SfMCT. (**a**) Time-dependent uptake of [^{14}C]L-lactate into SfMCT-overexpressing and vector-transformed bacteria (left). Net transport of [^{14}C]L-lactate shows saturation after 60 min (right). Data are represented as mean ± SEM from a representative triplicate experiment. (**b**) Evaluation of selected radiolabeled monocarboxylates (i.e., 0.1 μCi of [^{14}C]L-lactate, [^{3}H]propionate, [^{3}H]butyrate, and [^{14}C]pyruvate) as potential SfMCT substrates. SfMCT: SfMCT-overexpressing bacteria. Vector: vector-transformed bacteria. The data indicate that SfMCT is highly selective for the substrate [^{14}C]L-lactate. Data are represented as mean ± SEM from a representative triplicate experiment. (**c**) Effect of 20 μM valinomycin on the uptake of [^{14}C]L-lactate into SfMCT-overexpressing bacteria. Data are represented as mean ± SEM from three independent experiments, each in triplicate. (**d**) pH-dependent uptake of [^{14}C]L-lactate into SfMCT-overexpressing bacteria. The trace represents the mean of three independent experiments (each in triplicate) that were individually normalized (see Methods section). Data are represented as mean ± SEM from three independent experiments, each in triplicate. If not visible, error bars are smaller than symbols. (**e**) Proton-coupled transport of L-lactate into SfMCT-overexpressing and vector-transformed bacteria measured by a micro pH electrode based assay[5,6]. Representative pH curves are shown. At time point "0 s", where the bacteria suspension had a pH of 6.7, L-lactate was added to a final concentration of 10 mM using a stock of 2 M L-lactate, 250 mM KCl, 1 mM $MgSO_4$, 2 mM $CaCl_2$, pH 6.5. Addition of L-lactate resulted in a net pH increase (i.e., removal of protons from the extracellular solution) reflecting SfMCT mediated co-transport of protons together with L-lactate from the extracellular solution into bacteria. (**f**) Micro pH electrode based assay to evaluate if TSA is a potential substrate of the proton-coupled transporter SfMCT using SfMCT-overexpressing and vector-transformed bacteria. Representative pH curves are shown. At time point "0 s" TSA was added to a final concentration of 10 mM using a stock of 1 M TSA, 250 mM KCl, 1 mM $MgSO_4$, 2 mM $CaCl_2$,

pH 6.5. In contrast to the addition of the substrate L-lactate (panel e), the pH-value of both bacterial suspensions dropped and recovered over time indicating that TSA is not a substrate of SfMCT, in line with previous published experiments using this assay[5]. (**g**) Competition by 1 mM ACAC of [^{14}C]L-lactate uptake through wild-type SfMCT (WT) and SfMCT-F335A. Data are represented as mean ± SEM from three independent experiments, each in triplicate. Source data are provided as a Source Data file.

Supplementary Figure 2 Molecular structures of the physiologically relevant competitors used to determine the substrate specificity of SfMCT

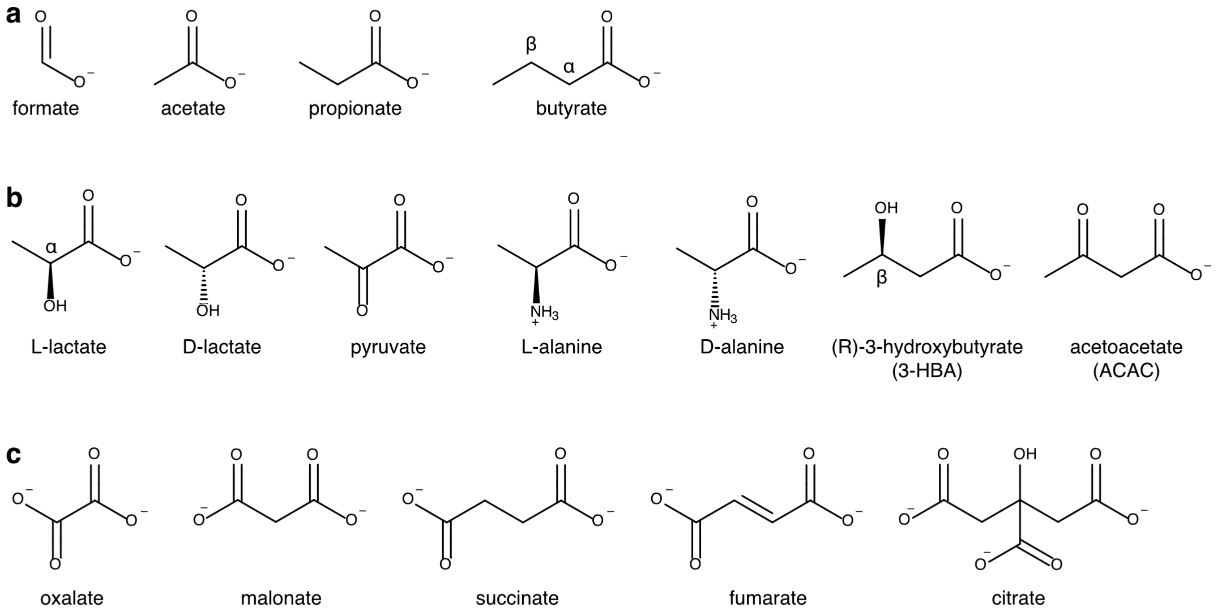

Supplementary Figure 2. Molecular structures of the physiologically relevant competitors used to determine the substrate specificity of SfMCT by [^{14}C]L-lactate uptake competition (Fig. 1b). (**a**) Linear unsubstituted monocarboxylates with one to four carbon atoms (i.e., formate to butyrate). (**b**) Monocarboxylates that are substituted at the α- or β-carbon atom. (**c**) Di- and tricarboxylates.

Supplementary Figure 3 Confirmation of the register of the built SfMCT model by selenomethionine SAD and alignment of the two monomers of the asymmetric unit

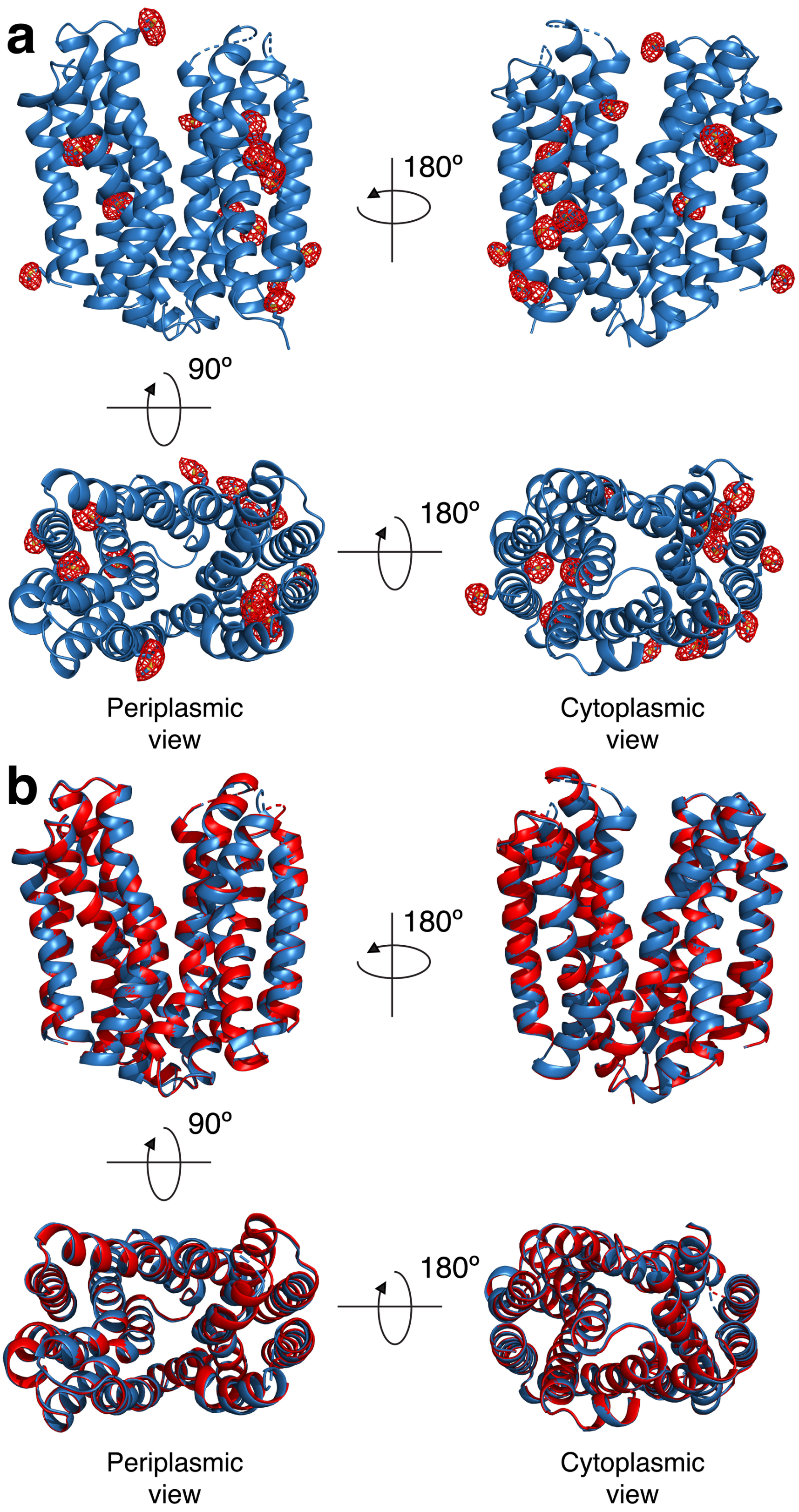

Supplementary Figure 3 Confirmation of the register of the built SfMCT model by selenomethionine SAD and alignment of the two monomers of the asymmetric unit. (**a**) Views onto the solved SfMCT structure (blue ribbon) combined with the anomalous difference electron densities (colored in red and contoured at 4 σ) obtained from SAD phasing of crystals grown from selenomethionine-substituted SfMCT. Methionine residues of SfMCT (shown as sticks) co-localize with the respective anomalous difference electron densities. Residues M1-T6 (N-terminus), L189 to W219 (loop between TM6 and TM7), and K405 to Q420 (C-terminus) are not shown. Broken lines show parts of loops that could not be traced. (**b**) The two monomers (in red and blue) of the asymmetric unit were superposed to each other using the align program that is implemented in PyMOL resulting in a root-mean-square deviation of 0.261 Å over 2,158 atoms. For the alignment M1-R10 (N-terminus), L189 to W219 (loop between TM6 and TM7), and K405 to Q420 (C-terminus) were removed.

Supplementary Figure 4 Crystal packing and contacts of SfMCT in space group *P*2_12_12

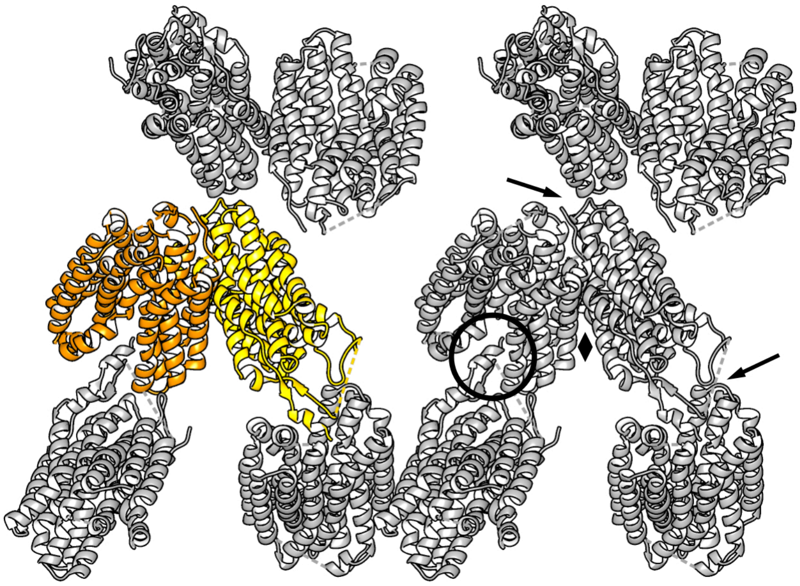

Supplementary Figure 4 Crystal packing and contacts of SfMCT in space group *P*2_12_12. In the asymmetric unit two SfMCT molecules make a crystal contact over TM12 (diamond). The loops between TM7 and TM8 of two SfMCT molecules of adjacent asymmetric units are involved in a second crystal contact (upper arrow). A third crystal contact is formed between the loop enclosed by TM6 and TM7, and the N-terminal end of TM10 (lower arrow). The C-terminus of one SfMCT molecule is located in the periplasmic cavity of another SfMCT of a neighboring asymmetric unit in a tongue-and-groove manner forming a fourth crystal contact (circle).

Supplementary Figure 5 Electron density and omit maps of ligand-bound SfMCT binding pockets

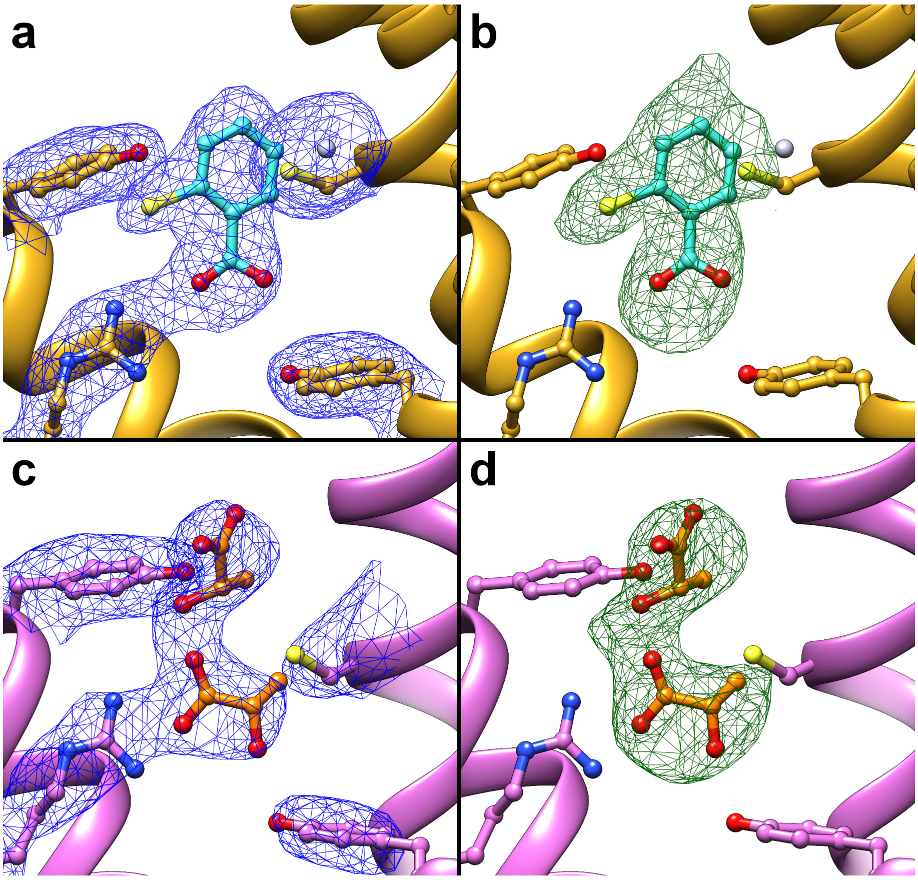

Supplementary Figure 5 Electron density and omit maps of ligand-bound SfMCT binding pockets. Electron density maps of the substrate-binding pockets with bound TSA ((**a**) and (**b**)), and L-lactate ((**c**) and (**d**)). The $2F_o$-F_c electron density maps ((**a**) and (**c**)) are contoured at 1.0 σ and colored in blue. The omit maps ((**b**) and (**d**)) are contoured at 3.0 σ and colored in green. The TSA- and L-lactate-bound SfMCT structures are represented as ribbons, and specific amino acid residues of the ligand-binding pockets are shown as ball-and-stick in yellow and magenta, respectively. The ligands TSA ((**a**) and (**b**)) and the two L-lactate molecules ((**c**) and (**d**)) are colored in cyan and orange, respectively. Mercury atoms in panels (**a**) and (**b**) are shown in grey.

Supplementary Figure 6 L-lactate binding to purified, detergent-solubilized SfMCT and SfMCT-R280A by microscale thermophoresis

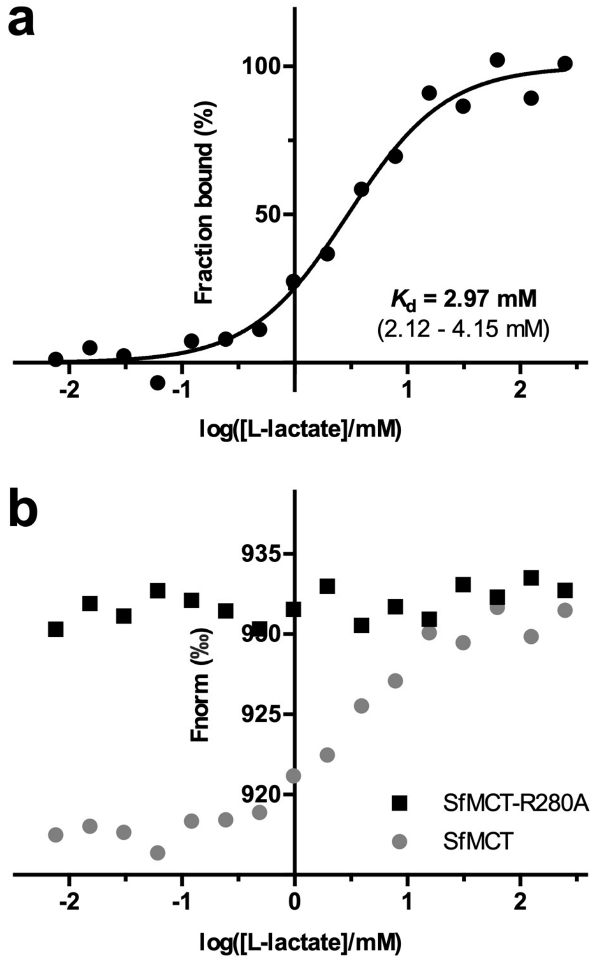

Supplementary Figure 6 L-lactate binding to purified, detergent-solubilized SfMCT and SfMCT-R280A by microscale thermophoresis. (**a**) A representative binding curve of L-lactate to SfMCT is shown. The solid line represents the fit of the mass action equation to the experimental data. An average K_d-value of 2.84 ± 0.15 mM (average ± SEM) was calculated from 5 independent experiments. (**b**) Data of a representative microscale thermophoresis experiment showing no binding of L-lactate to SfMCT-R280A. For comparison the normalized fluorescence signal (Fnorm) of L-lactate binding to SfMCT is also shown. Source data are provided as a Source Data file.

Supplementary Figure 7 Western blot analysis of SfMCT wild-type and mutants

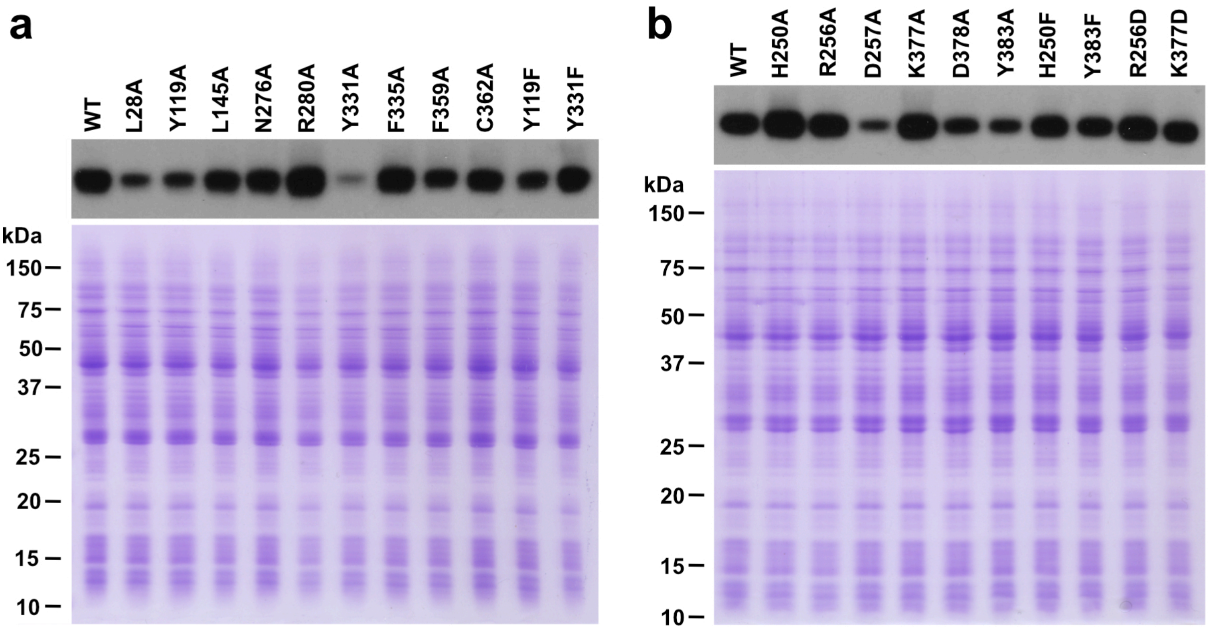

Supplementary Figure 7 Western blot analysis of SfMCT wild-type and mutants. Western blot (top) detection of the expression of (**a**) SfMCT ligand binding pocket mutants and (**b**) SfMCT proton binding site mutants in *E. coli* JA202[7] using an anti-His_5 and goat anti-mouse IgG (H+L) HRP conjugate antibody. Equal amounts of bacteria expressing SfMCT variants were loaded on 14% SDS/polyacrylamide gels. The Coomassie-stained 14% SDS/polyacrylamide gels (bottom) served as loading controls for Western blot analysis. The same amount of lyzed bacteria were analyzed on the Western blot and the Coomassie-stained gels. Similar intensities of the Coomassie-stained gels show that similar amounts of lyzed bacteria expressing SfMCT variants were loaded on the gels. Source data are provided as a Source Data file.

Supplementary Figure 8 Multiple sequence alignment of SfMCT and other bacterial transporters, and molecular interactions between R280 and N276 of SfMCT

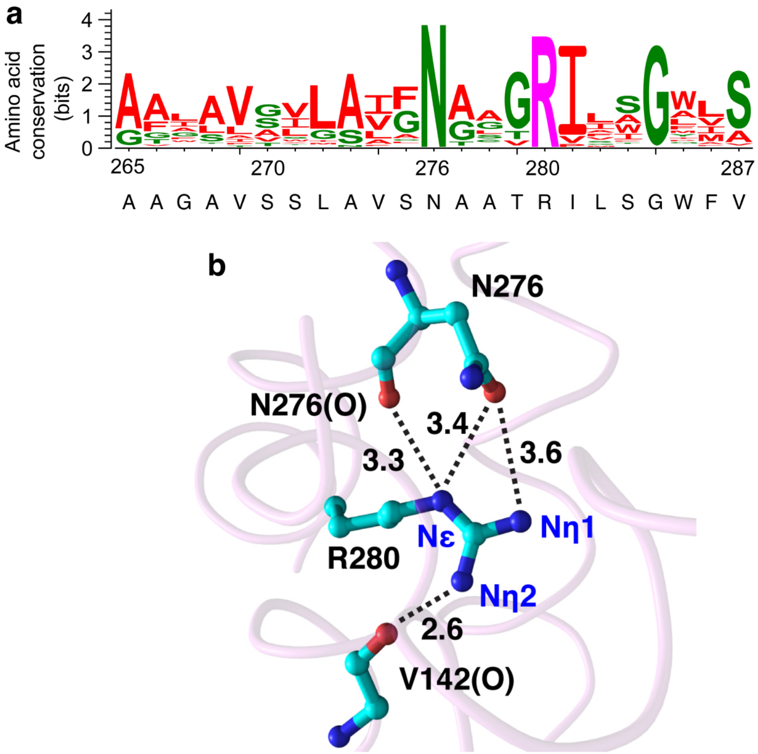

Supplementary Figure 8 Multiple sequence alignment of SfMCT and other bacterial transporters, and molecular interactions between R280 and N276 of SfMCT. (**a**) A sequence logo representation of the region close (+/- 11 amino acids) to N276 of SfMCT using a multiple sequence alignment of SfMCT and bacterial homologues is shown. Color-coding of amino acid residues is according to their physicochemical properties, i.e., small and hydrophobic (red), basic (magenta) and other (green) amino acid residues. The amino acid sequence of the corresponding SfMCT region is shown below the sequence logo representation. (**b**) N276 interacts with the guanidinium group of R280 via hydrogen bonds between its side chain amide group and the backbone carbonyl oxygen atom (N276(O) (TM8)), and the Nε and Nη1 nitrogen atoms of the guanidinium group of R280 (TM8). In addition, the Nη2 nitrogen atom of the guanidinium group of R280 (TM8) is hydrogen bonded to the backbone carbonyl oxygen of V142 (V142(O) (TM5)). Corresponding amino acids are shown as ball-and-stick in cyan. Distances are given in Ångström (Å).

Supplementary Figure 9 Multiple sequence alignment of SfMCT and human MCT1-4

```
                                             TM 1
SfMCT      -----MADQ------QTTMPRWVPLLLGLLGSTTCGMLLYAWSVFIKPLNAEFGWSRAEI    49
hMCT1      --MPPAVGGPVGYTPPDGGWGWAVVIGAFISIGFSYAFPKSITVFFKEIEGIFHATTSEV    58
hMCT2      --MPPMPSAPPVHPPPDGGWGWIVVGAAFISIGFSYAFPKAVTVFFKEIQQIFHTTYSEI    58
hMCT3      MGA---GGPRRGEGPPDGGWGWVVLGACFVVTGFAYGFPKAVSVFFRALMRDFDAGYSDT    57
hMCT4      MGGAVVDEGPTGVKAPDGGWGWAVLFGCFVITGFSYAFPKAVSVFFKELIQEFGIGYSDT    60
                                *  :   ::    .  :  : :**:: :   *    ::

                        TM 2                     TM 3
SfMCT      AMAFAICCLIFGLMTFPA----GRLSDKMGPRKVVMTGGVLLAIGFILSGFIQSKYQLYI    105
hMCT1      SWISSIML----AVMYGGGPISSILVNKYGSRIVMIVGGCLSGCGLIAASFCNTVQQLYV    114
hMCT2      AWISSIML----AVMYAGGPVSSVLVNKYGSRPVVIAGGLLCCLGMVLASFSSSVVQLYL    114
hMCT3      AWVSSIML----AMLYGTGPVSSILVTRFGCRPVMLAGGLLASAGMILASFATRLLELYL    113
hMCT4      AWISSILL----AMLYGTGPLCSVCVNRFGCRPVMLVGGLFASLGMVAASFCRSIIQVYL    116
           :   :*       : :      .    : * * *::.** :   *:: :.*     ::*:

                    TM 4                            TM 5
SfMCT      TYGVIAGFGGGMIYLPPIATAPKWWPDRRALATGFAVVGLGLGSFLMGPLATYIIEKPGM    165
hMCT1      CIGVIGGLGLAFNLNPALTMIGKYFYKRRPLANGLAMAGSPVFLCTLAPLNQVFFGI--F    172
hMCT2      TMGFITGLGLAFNLQPALTIIGKYFYRKRPMANGLAMAGSPVFLSSLAPFNQYLFNT--F    172
hMCT3      TAGVLTGLGLALNFQPSLIMLGLYFERRRPLANGLAAAGSPVFLSALSPLGQQLLER--F    171
hMCT4      TTGVITGLGLALNFQPSLIMLNRYFSKRRPMANGLAAAGSPVFLCALSPLGQLLQDR--Y    174
             *.: *:* .:   * :     ::  :* :*.*:* .*  :    :.*:   :

                      TM 6
SfMCT      GWRYVFWYCGVAMGIMALIAGAFLEPPPAGWKPAGYTPPA---PPAGAAAPK--------    214
hMCT1      GWRGSFLILGGLL-LNCCVAGALMRPIGPKPTKAGKDKSKASLEKAGKSGVKKDLHDANT    231
hMCT2      GWKGSFLILGSLL-LNACVAGSLMRPLGPNQTTSKSKNKTGKTE----------------    215
hMCT3      GWRGGFLLLGGLL-LHCCACGAVMRPPPGPGPRPRRDSAG---DRAGDAPGEAEA-----    222
hMCT4      GWRGGFLILGGLL-LNCCVCAALMRPLVVTAQ----------------------------    205
           **:  *   *  : : .  ..:.:.*

                                                     TM 7
SfMCT      --------VTR--DWTY-----EEAKGDTKFWLLYLAYFCGSFAGL-MVIGHLAGFGRDA    258
hMCT1      DLIGRHPKQEKRSVFQTINQFLDLTLFTHRGFLLYLSGNVIMFFGLFAPLVFLSSYGKSQ    291
hMCT2      DDSSPKKIKTKKSTWEKVNKYLDFSLFKHRGFLIYLSGNVIMFLGFFAPIIFLAPYAKDQ    275
hMCT3      DG-AGLQLREASPRVRPRRRLLDLAVCTDRAFAVYAVTKFLMALGLFVPAILLVNYAKDA    281
hMCT4      ---------PGSGPPRPSRRLLDLSVFRDRGFVLYAVAASVMVLGLFVPPVFVVSYAKDL    256
                                 : :    : : :*         *:      :  :.:.

                        TM 8                          TM 9
SfMCT      GLTAMAAAGAVSSLAFSNAATRILSGWFVDKIGIRVYFAALFALQTAA---MIAIFQLGG    315
hMCT1      HYSSEKSAFLLSILAFVDMVARPSMGLVANTKPIRPRIQYFFAASVVANGVCHMLAPLST    351
hMCT2      GIDEYSAAFLLSVMAFVDMFARPSVGLIANSKYIRPRIQYFFSFAIMFNGVCHLLCPLAQ    335
hMCT3      GVPDTDAAFLLSIVGFVDIVARPACGALAGLARLRPHVPYLFSLALLANGLTDLSSARAR    341
hMCT4      GVPDTKAAFLLTILGFIDIFARPAAGFVAGLGKVRPYSVYLFSFSMFFNGLADLAGSTAG    316
                 :*  :: :.* :  :*   * ...   :*     :*:               .

                        TM 10                           TM 11
SfMCT      SVVGLSIVAIVIGWNYGAMFTLFPATCLQFYGPTAQGSNYGLLFTACGLAGFAGPWVGGW    375
hMCT1      TYVGFCVYAGFFGFAFGWLSSVLFETLMDLVGPQRFSSAVGLVTIVECCPVLLGPPLLGR    411
hMCT2      DYTSLVLYAVFFGLGFGSVSSVLFETLMDLVGAPRFSSAVGLVTIVECGPVLLGPPLAGK    395
hMCT3      SYGALVAFCVAFGLSYGMVGALQFEVLMAAVGAPRFPSALGLVLLVEAAAVLIGPPSAGR    401
hMCT4      DYGGLVVFCIFFGISYGMVGALQFEVLMAIVGTHKFSSAIGLVLLMEAVAVLVGPPSGGK    376
              .:   .  :*  :* : ::   . :   *     *  **:        : **   *

                             TM 12
SfMCT      LKDTTGTYYLPFLCAAALCAL-------GTAIVFMTKPPEKKHA----------------    412
hMCT1      LNDMYGDYKYTYWACGVVLIISGIYLFIGMGINYRLLAKEQKANEQKKESKEEETSIDVA    471
hMCT2      LVDLTGEYKYMYMSCGAIVVAASVWLLIGNAINYRLLAKERKEENARQKTRESEPLSKSK    455
hMCT3      LVDVLKNYEIIFYLAGSEVALAGVFMAVATNCCLRCAKAAPSGPGTEGGASDTEDAE-AE    460
hMCT4      LLDATHVYMYVFILAGAEVLTSSLILLLGNFFCIRKKPKE---PQPEVAAAEEEKLH-KP    432
           * *    *    :  ..            .

SfMCT      ---------------------------------------------     412
hMCT1      GKPNEVTKAAESPDQKDTD------GGPKEEES---PV------     500
hMCT2      -HSEDVNVKVSN-AQ----------SVTSERET---NI------     478
hMCT3      GDSEPLPVVAEEPGNLEALEVLSARGEPTEPEIEARPRLAAESV     504
hMCT4      PAD--------SGVDLREVEHFLKAEPEKNGEVVHTPETSV---     465
```

Supplementary Figure 9 Multiple sequence alignment of SfMCT and human MCT1-4. Amino acid sequence alignment was performed with Clustal Omega[8]. The UniProt ID codes of SfMCT, human MCT1 (hMCT1), human MCT2 (hMCT2), human MCT3 (hMCT3) and human MCT4 (hMCT4) are A0LNN5, P53985, O60669, O95907 and O15427, respectively. Positions that have a single, fully conserved residue are indicated by (*), conservation between groups of strongly and weakly similar properties are indicated by (:) and (·), respectively. Color-coding of amino acid residues is according to their physicochemical properties, i.e., small and hydrophobic (red), acidic (blue), basic (magenta) and other (green) amino acid residues. The positions of transmembrane helices according to the SfMCT structure are highlighted in grey. Residues involved in ligand binding are highlighted in yellow. Proton binding site residues are highlighted in cyan.

Supplementary Figure 10 Electrostatic surface potential of SfMCT variants

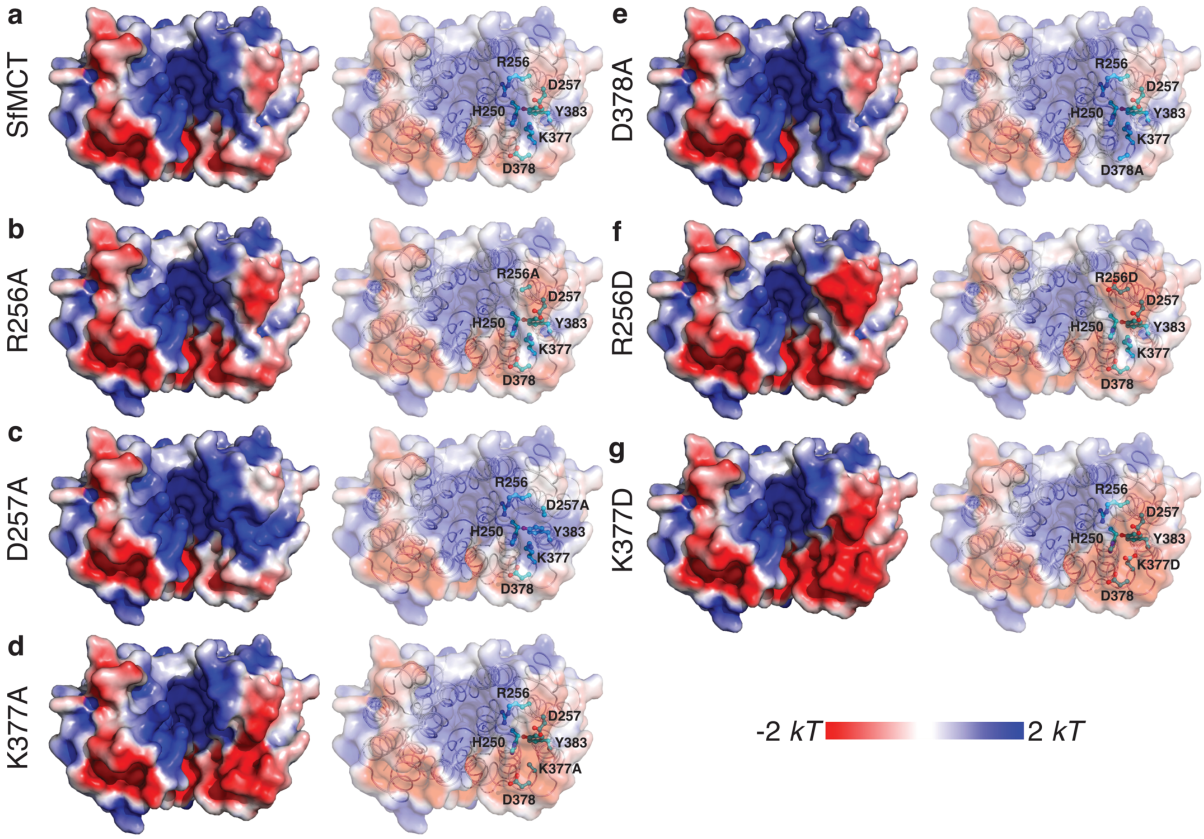

Supplementary Figure 10 Electrostatic surface potential of SfMCT variants. The displayed electrostatic surface potentials of wild-type SfMCT (SfMCT) (**a**) and proton binding site region mutants R256A (**b**), D257A (**c**), K377A (**d**), D378A (**e**), R256D (**f**) and K377D (**g**) were calculated by the Adaptive Poisson-Boltzmann Solver (APBS)[9]. The maps are color coded from negative (red) to neutral (white) to positive (blue) surface potentials.

Supplementary Figure 11 Comparison of SfMCT and rat MCT1 homology model

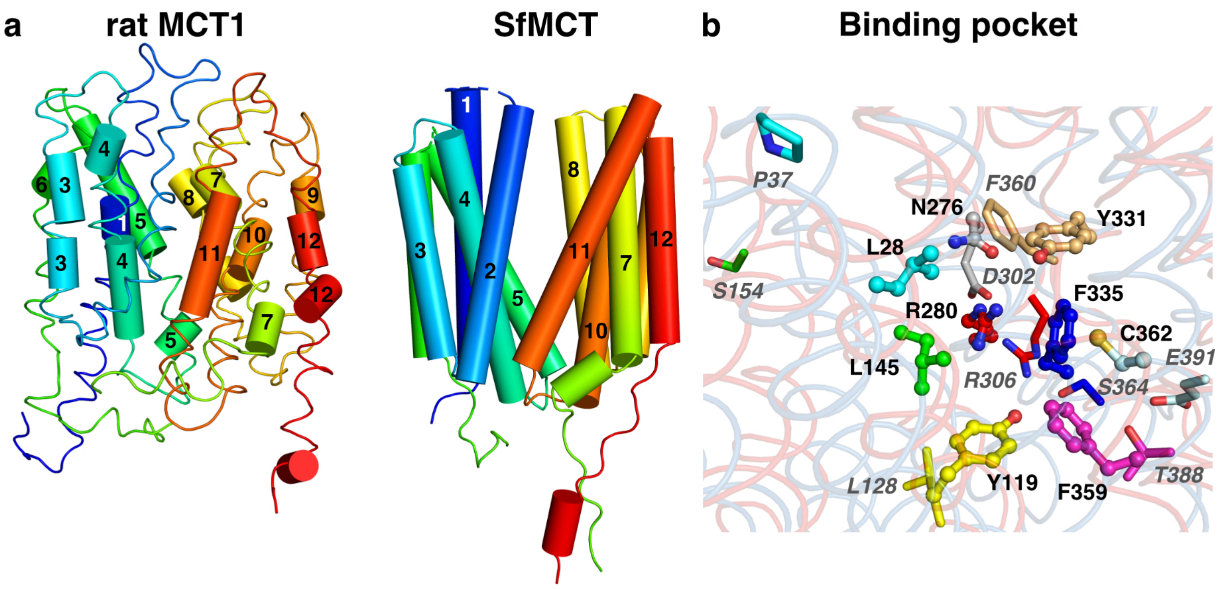

Supplementary Figure 11 Comparison of the outward-open SfMCT structure and the outward-open rat MCT1 homology model. (**a**) Presentation of the secondary structure elements of the outward-open rat MCT1 homology model[10] (left) and the experimental SfMCT structure (right). The secondary structure elements were determined using the dss algorithm implemented in PyMOL. The secondary structure information of the SfMCT structure file was removed for unbiased analysis. The rat MCT1 homology model and the SfMCT structure were aligned using the cealign algorithm implemented in PyMOL and separated by horizontal translation. (**b**) Comparison of SfMCT ligand binding site residues (ball-and-stick representation) with the corresponding residues in the outward-open rat MCT1 homology model (stick representation). Corresponding residues have the same color. Residue labeling is in black for SfMCT, and gray and italic for rat MCT1. The cartoon representation of the SfMCT and rat MCT1 models are in bright blue and bright red, respectively.

Supplementary References

1. Evans, P. R. & Murshudov, G. N. How good are my data and what is the resolution? *Acta Crystallogr. D Biol. Crystallogr.* **69,** 1204–1214 (2013).
2. Diederichs, K. & Karplus, P. A. Improved R-factors for diffraction data analysis in macromolecular crystallography. *Nat. Struct. Biol.* **4,** 269–275 (1997).
3. Weiss, M. Global indicators of X-ray data quality. *J. Appl. Cryst.* **34,** 130–135 (2001).
4. Karplus, P. A. & Diederichs, K. Linking crystallographic model and data quality. *Science* **336,** 1030–1033 (2012).
5. Franco, P. J. & Brooker, R. J. Functional roles of Glu-269 and Glu-325 within the lactose permease of *Escherichia coli*. *J. Biol. Chem.* **269,** 7379–7386 (1994).
6. Harder, D. *et al.* Engineering a chemical switch into the light-driven proton pump proteorhodopsin by cysteine mutagenesis and thiol modification. *Angew. Chem. Int. Ed.* **55,** 8846–8849 (2016).
7. Núñez, M. F., Pellicer, M. T., Badía, J., Aguilar, J. & Baldomà, L. The gene *yghK* linked to the *glc* operon of *Escherichia coli* encodes a permease for glycolate that is structurally and functionally similar to L-lactate permease. *Microbiology* **147,** 1069–1077 (2001).
8. Sievers, F. *et al.* Fast, scalable generation of high-quality protein multiple sequence alignments using Clustal Omega. *Mol. Syst. Biol.* **7,** 539–539 (2011).
9. Baker, N. A., Sept, D., Joseph, S., Holst, M. J. & McCammon, J. A. Electrostatics of nanosystems: application to microtubules and the ribosome. *Proc. Natl. Acad. Sci. U S A* **98,** 10037–10041 (2001).
10. Wilson, M. C., Meredith, D., Bunnun, C., Sessions, R. B. & Halestrap, A. P. Studies on the DIDS-binding site of monocarboxylate transporter 1 suggest a homology model of the open conformation and a plausible translocation cycle. *J. Biol. Chem.* **284,** 20011–20021 (2009).
