# Supplementary material for: Mechanistic basis of L-lactate transport in the SLC16 solute carrier family
Source: Nat Commun. 2019 Jun 14;10:2649. doi: 10.1038/s41467-019-10566-6 (PMC6573034; doi:10.1038/s41467-019-10566-6)
Supplement: Supplementary file 3 — Reporting Summary [file 41467_2019_10566_MOESM3_ESM.pdf]

nature research

Corresponding author(s): Prof. Dr. Dimitrios Fotiadis

Reporting Summary

Nature Research wishes to improve the reproducibility of the work that we publish. This form provides structure for consistency and transparency in reporting. For further information on Nature Research policies, see Authors & Referees and the Editorial Policy Checklist.

Statistical parameters

When statistical analyses are reported, confirm that the following items are present in the relevant location (e.g. figure legend, table legend, main text, or Methods section).

n/a	Confirmed	
☐	☒	The exact sample size (n) for each experimental group/condition, given as a discrete number and unit of measurement
☐	☒	An indication of whether measurements were taken from distinct samples or whether the same sample was measured repeatedly
☒	☐	The statistical test(s) used AND whether they are one- or two-sided<br>*Only common tests should be described solely by name; describe more complex techniques in the Methods section.*
☒	☐	A description of all covariates tested
☒	☐	A description of any assumptions or corrections, such as tests of normality and adjustment for multiple comparisons
☒	☐	A full description of the statistics including central tendency (e.g. means) or other basic estimates (e.g. regression coefficient) AND variation (e.g. standard deviation) or associated estimates of uncertainty (e.g. confidence intervals)
☒	☐	For null hypothesis testing, the test statistic (e.g. F, t, r) with confidence intervals, effect sizes, degrees of freedom and P value noted<br>*Give P values as exact values whenever suitable.*
☒	☐	For Bayesian analysis, information on the choice of priors and Markov chain Monte Carlo settings
☒	☐	For hierarchical and complex designs, identification of the appropriate level for tests and full reporting of outcomes
☒	☐	Estimates of effect sizes (e.g. Cohen's d, Pearson's r), indicating how they were calculated
☐	☒	Clearly defined error bars<br>*State explicitly what error bars represent (e.g. SD, SE, CI)*

Our web collection on statistics for biologists may be useful.

Software and code

Policy information about availability of computer code

Data collection	X-ray datasets were collected at the X06SA (PXI) beamline of the Swiss Light Source (Paul Scherrer Institute, Villigen, Switzerland) using an EIGER 16M detector (Dectris).

Data analysis

Structure determination:
Datasets were indexed and integrated with XDS and merged using BLEND of the CCP4 program suite. Scaling and averaging of symmetry-related intensities were performed by aP_scale with truncation of the data at the best high-resolution along h, k or l axis determined by AIMLESS. The STARANISO software (http://staraniso.globalphasing.org/) was applied to account for the anisotropy of the data. The SAD method was applied using the CRANK2 pipeline running with SHELX/D, REFMAC5, Parrot and Buccaneer in the CCP4 program suite. To obtain a final structure, iterative refinement and model building were performed by phenix.refine and Coot, respectively. The structure of bMCT with bound L-lactate was obtained by molecular replacement with Phaser using the bMCT structure with bound TSA as search model. The final structures were obtained after multiple rounds of model building with Coot and refinement with phenix.refine. For all the refinements, XYZ coordinates, individual B-factors, occupancies and TLS strategies were applied. The TLS groups were automatically assigned using Phenix. Figures representing structural information were prepared using Chimera or PyMol. Van der Waals pore radii were computed using HOLE. Electrostatic surface potentials were calculated by the Adaptive Poisson-Boltzmann Solver (APBS).

Analysis of transport assays:
Data of transport assays were analyzed using Prism 6.

For manuscripts utilizing custom algorithms or software that are central to the research but not yet described in published literature, software must be made available to editors/reviewers upon request. We strongly encourage code deposition in a community repository (e.g. GitHub). See the Nature Research guidelines for submitting code & software for further information.

Data

Policy information about availability of data

All manuscripts must include a data availability statement. This statement should provide the following information, where applicable:

- Accession codes, unique identifiers, or web links for publicly available datasets
- A list of figures that have associated raw data
- A description of any restrictions on data availability

Atomic models have been deposited in the Protein Data Bank under accession numbers 6G9X and 6HCL.

Field-specific reporting

Please select the best fit for your research. If you are not sure, read the appropriate sections before making your selection.

☒ Life sciences ☐ Behavioural & social sciences ☐ Ecological, evolutionary & environmental sciences

For a reference copy of the document with all sections, see nature.com/authors/policies/ReportingSummary-flat.pdf

Life sciences study design

All studies must disclose on these points even when the disclosure is negative.

Sample size	For transport studies, presented data originate from 3 independent experiments, each performed in triplicates.
Data exclusions	In transport studies, no data was excluded from the analysis.
Replication	Transport experiments were independently repeated 3 times.<br>For X-ray crystallography experiments more >1000 crystals were measured.
Randomization	Randomization is not necessary and was thus not applied.
Blinding	Blinding was not applied because it is not necessary in the field of X-ray crystallography.

Reporting for specific materials, systems and methods

Materials & experimental systems

n/a	Involved in the study	
☒	☐	Unique biological materials
☐	☒	Antibodies
☒	☐	Eukaryotic cell lines
☒	☐	Palaeontology
☐	☒	Animals and other organisms
☒	☐	Human research participants

Methods

n/a	Involved in the study	
☒	☐	ChIP-seq
☒	☐	Flow cytometry
☒	☐	MRI-based neuroimaging

Antibodies

Antibodies used	Commercial anti-pentaHis (Qiagen, catalogue number 34660) and goat anti-mouse IgG (H+L) HRP conjugate antibody (Biorad, catalogue number 172-1011).
Validation	Commercial antibodies

Animals and other organisms

Policy information about studies involving animals; ARRIVE guidelines recommended for reporting animal research

Laboratory animals	Escherichia coli BL21(DE3) pLysS, Escherichia coli JA202
Wild animals	The study did not involve wild animals.
Field-collected samples	The study did not involve any field-collected samples.
